# Phenotype versus genotype to optimize cancer dosing in the clinical setting—focus on 5‐fluorouracil and tyrosine kinase inhibitors

**DOI:** 10.1002/prp2.1182

**Published:** 2024-03-01

**Authors:** Jennifer H. Martin, Peter Galettis, Alex Flynn, Jennifer Schneider

**Affiliations:** ^1^ Drug Repurposing and Medicines Research Program Hunter Medical Research Institute New Lambton Heights New South Wales Australia

**Keywords:** dose individualization, genotype, phenotype, therapeutic drug monitoring

## Abstract

Cancer medicines often have narrow therapeutic windows; toxicity can be severe and sometimes fatal, but inadequate dose intensity reduces efficacy and survival. Determining the optimal dose for each patient is difficult, with body‐surface area used most commonly for chemotherapy and flat dosing for tyrosine kinase inhibitors, despite accumulating evidence of a wide range of exposures in individual patients with many receiving a suboptimal dose with these strategies. Therapeutic drug monitoring (measuring the drug concentration in a biological fluid, usually plasma) (TDM) is an accepted and well validated method to guide dose adjustments for individual patients to improve this. However, implementing TDM in routine care has been difficult outside a research context. The development of genotyping of various proteins involved in drug elimination and activity has gained prominence, with several but not all Guideline groups recommending dose reductions for particular variant genotypes. However, there is increasing concern that dosing recommendations are based on limited data sets and may lead to unnecessary underdosing and increased cancer mortality. This Review discusses the evidence surrounding genotyping and TDM to guide decisions around best practice.

## Graphical abstract

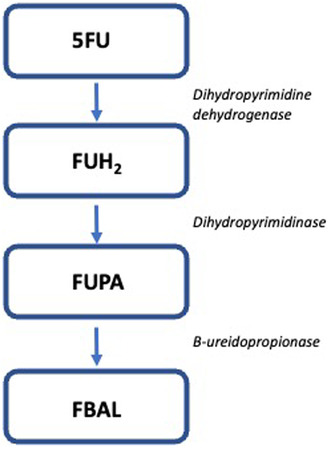

## 1 INTRODUCTION

For 50 years, therapeutic drug monitoring (TDM), which involves measuring drug concentrations in plasma or whole blood and adjusting the dose to optimize clinical management, has been available to individualize medicine dosing. In the oncology setting, TDM is an attractive option as the commonly used methods of cancer dosing, using body surface area, or fixed flat dosing do not always result in attaining the desired concentrations in many patients. A recent prospective study with 5‐fluorouracil (5FU) reported that only 20.3% of patients dosed based on body surface area achieved the target area under the curve (AUC) values with 19.1% above the target range, and 60.6% were underdosed.^1^ There is similarly wide interindividual variability in the pharmacokinetics of tyrosine kinase inhibitors (TKIs) with fixed flat dosing.^2^

Most cancer medicines have narrow therapeutic windows. Toxicity can be severe and sometimes fatal, but inadequate exposure reduces survival outcomes.^3^ Therefore, understanding the drug exposure for a particular patient is paramount. TDM is increasingly proposed in the literature as an approach to selecting optimal doses for oral anticancer drugs.^4^ The feasibility of the clinical application of TDM‐guided precision dosing to oral anticancer drugs was demonstrated in a European prospective multicenter study^5^ and a similar study is underway in the Netherlands.^6^ However, the routine use of TDM outside research laboratories or feasibility trials appears to be limited overall.

Performing TDM requires a multidisciplinary approach, usually comprised of scientists and clinicians including clinical pharmacologists, nurses, and pharmacists—particularly those skilled in predicting drug exposure at different doses. TDM consists of the following: a clinician makes a diagnosis and chooses a drug for which TDM has shown to improve outcomes; measurement is taken at the correct time (usually a trough, at steady state); clinical assessments of the drug efficacy and safety are made; drug measurement is undertaken using a chromatography system (depending on the drug and access to measurement resources); a pharmacokinetic model or algorithm is used (with clinical judgment), and a decision around increasing or decreasing dosing is made. Genetic testing, however, can be done at any time of the patient's journey, requires a genetic test, and the clinician can use that to help guide choice of a drug (and sometimes dose).

A search of pathology tests available across Australian pathology services by the authors identified no state services offering testing for 5FU or targeted therapies with the exception of imatinib. This lack of uptake is compounded by sampling times requiring an extra pathology visit; transporting samples to the laboratory in a timely fashion; integrating them with standard laboratory practices; and enabling adequate funding to cover not just the tests but the clinical pharmacological interpretation and dose adjustment recommendation.^7^

Alongside TDM is the development of pharmacogenetics. Here a gene encoding an enzyme critical to drug metabolism is identified. Genetic variation is shown to affect enzymatic activity, usually resulting in reduced enzymatic activity and toxicity for the patient. This is especially problematic in homozygous or compound heterozygous variants where enzymatic activity is critically low, potentially causing life‐threatening toxicity. Identifying these patients before they receive any of the associated anti‐neoplastic medicine should be a matter of priority. More complicated, however, is the group of patients who have a variant where the clinical implication is unknown, based on a small dataset, or only described in one ethnic population, and the decision for the treating oncologist is between avoiding a potential and rare risk of toxicity and avoiding a more common risk of underdosing and reduced efficacy.

Genetic testing measures a partial or absent form of a gene coding for an enzyme involved in metabolism to a major contributing metabolite or action, such as dihydropyridine deficiency (DPD), which is the rate‐limiting step for 5‐FU metabolism. If present, and depending on whether the patient is homozygous, or heterozygous for one of the four common variants, the dose is arbitrarily reduced (by a third, half, or not given, e.g., as recommended by CPIC^8^, ^9^ and in the guidance from cancer centers such as Peter MacCallum Cancer Institute for 5‐FU). This dose reduction is predicted by the likely subsequent activity of a reduced gene expression, even when this relationship is loose, complex, or unclear.

Dose changes based on the selection of gene mutations identified can prevent much but not all serious morbidity. However, accurate and individualized dose adjustment based on the genetic result is complex to perform. This is because there are still significant adverse events occuring even in the absence of gene mutations. This is likely to be due to epigenetic events or to the gene or additional genes involved in drug activity or metabolism that have not yet been characterized, or other factors causing changes in drug metabolism in genes that are not measured. These cases can lead to underdosing, therefore dosing by the limited gene test is not dose optimizing. In many cases, significant research and government funding have resulted in the implementation of genetic testing into clinical practice being ahead of the clinical implementation of TDM, and further, genetic testing has been integrated into some (but not all) international guidelines.^10^, ^11^ Genotyping uses a broad categorization approach for dosing advice and may appear more straightforward than TDM which uses predictive modeling for dose adjustment. However, it is notable that the Federal Drugs Agency, the US regulatory authority, in contradistinction, has recently set up a project to investigate. “Project Optimus”^12^ is an FDA oncology center of excellence initiative to improve the dose optimization and dose selection paradigm in oncology drug development.

The risks of underdosing are of increasing concern. They are highlighted in the International Association of Therapeutic Drug Monitoring and Clinical Toxicology (IATDMCT) summary,^30^ the lower achievement of target AUC using BSA,^13^, ^14^ and discussed at two recent international meetings.^15^, ^16^

Although optimal dosing may include consideration of multiple factors, the scientific literature has become increasingly divided into whether phenotype (TDM or other phenotype measures, e.g., measuring plasma uracil as a marker of DPD activity for 5‐FU) or metabolic genotype (genes that metabolize drugs) is the most clinically relevant approach for individualized dosing. The awareness of how such division (genetics vs. cancer biology vs. pharmacology) is not conducive to optimal patient care has arguably been missing in this discussion. However, Implementing both genotype and phenotype aspects of individualized dosing is important to patient care as from a clinical perspective putting the patient first is key. Weighing up the relative risk and benefit of both TDM or genotype to guide the decision on the dosing of each drug should be encouraged.

This Short Review covers a narrative discussion on this area, using general examples of 5‐FU and tyrosine kinase inhibitor (TKI) dosing for illustration. Reviews of the scientific evidence for TDM in cancer are large and many, and the authors direct the audience to such reviews.^17^, ^18^ The opinions of the authors are from experience in clinical pharmacology, medical oncology, and laboratory cancer pharmacology together with discussions after recent presentations at international meetings. The literature review consisted of a narrative review of current global guidelines and contributing evidence, clinical trial evidence to support the guidelines, and current clinical practice.^9^, ^10^, ^11^ Two chemotherapy medications with a number of both genetic and phenotypic variables were chosen in order to illustrate this clinical practice issue (Tables 1 and 2).

**TABLE 1:** 5FU pharmacogenetic testing.

Facts and issues that support gene testing to guide dose	Facts and issues that do not support gene testing to guide dose
There is a wide range of DPD expression between individuals.	DPD activity may be more clinically helpful than DPD expression.
The four major polymorphisms in the DPD gene that may result in partial or total loss of DPD activity have now been identified.	There is not a linear correlation between polymorphism and DPD activity. Assuming such leads to underdosing which could increase mortality..
The incidence of DPD deficiency depends on the technique used to screen and the population which is studied.	Clinical decisions may be made on incomplete science.
3%–8% of the European population have DPD deficiency correlating with up to 50% less enzyme activity, and 0.1% are estimated to have complete DPD deficiency.	Cost‐effectiveness of toxicity prevention in the total cancer population having 5FU is unclear. However, it is noted that the risk could be catastrophic for the 0.1% if dose is not withheld or reduced. The frequency of DPD alleles is also different in non‐Caucasian populations.
Gene expression is a numerical value.	Quantification of dose reduction is unclear, however.
20% 5FU catabolized by other pathways	Dose reduction for some gene variants may lead to underdosing
	As well as the four "common" polymorphic genetic variants of DPD, there are other relevant enzymes which are polymorphic e.g., methylene tetra‐hydro‐folate reductase. The c.C677T polymorphism can cause a 30% reduction in enzymatic activity leading to an accumulation of 5,10‐methylene‐THF and increased risk of grade 3/4 toxicity^15^
*DPD is relatively specific for pyrimidine metabolism*.	Genes other than metabolic ones can be responsible for 5FU toxicity, e.g., drug transporters, e.g., by ABC1 may be important in activity and resistance, evidenced by enhanced ABC5 expression in colorectal cancer patients after 5‐FU‐based chemotherapy. ABCB11 is involved in efflux transport of FdUMP (reviewed in 15).
DPD testing can enable a reduction in dose before the first script and before AEs occur.	The genotype is not always correlated with phenotype, and underdosing may occur. Pre‐emptive screening may cause a delay in treatment.
Cost savings for reduced AEs and need for reversal agents, e.g., uridine triacetate	Cost of screening for a 0.1% genotype is high. From an equity perspective, most around the world will not be able to pay, and in wealthier jurisdictions, insurance companies may not cover the cost of DPD genetic testing.

**TABLE 2:** 5FU phenotyping testing.

Facts and issues that support phenotype testing to guide dose	Facts and issues that do not support phenotype testing to guide dose
Measurement of uracil concentration or dihydrouracil:uracil ratio (UH2/U) is a measure of individual DPD activity.	Patients with DPD deficiency do not always have a UH2/U ratio below the "normal" threshold. The association between uracil levels and DPD activity is not clear.
Variability in the genotype–phenotype relationship can be due to post‐transcriptional regulation of DPD via short RNAs associated with RNA‐induced silencing complex proteins binding to DPD mRNA to inhibit translation and increase degradation. Post‐transcriptional regulation of DPD involves microRNAs (miR‐27a and miR‐27b).	Inappropriate dosing decisions can be made if the phenotype is not correctly ascertained.
Not all carriers of DPD decreased/no function variants develop severe toxicity at standard doses.^17^, ^18^ Similarly, patients without a DPD decreased/no function variant may still experience severe toxicity due to different genetic, environmental, or other factors, such as diet (noting that 5FU resembles the pyrimidine base uracil).^19^, ^20^, ^21^	A phenotype measure needs to be validated to ensure DPYD status is not relied on to ensure that underdosing does not occur. Predicting phenotype (and plasma concentrations from a set dose) can be difficult with a drug that displays nonlinear pharmacokinetics such as 5FU, so measurements as the dose or physiology alters, need to be made.

### 1.1 5‐Flurouracil

**FIGURE 1:**
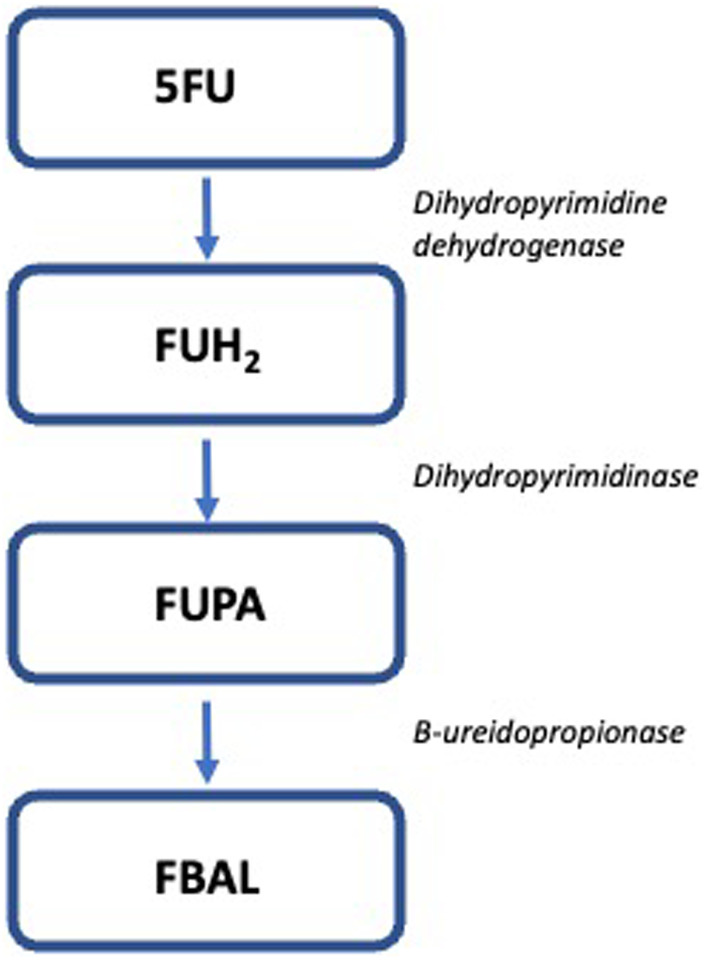
Metabolism of 5FU. FBAL, fluoro‐β‐alanine; FUH_2_, dihydrofluorouracil; FUPA, fluoro‐β‐ureidopropionic acid.

5‐Fluorouracil (5FU) was identified as an antimetabolite chemotherapy in 1957 and approved by the FDA in 1962 initially for the treatment of colorectal cancer. This mode of action was based on the observation that tumors used up uracil more rapidly than normal tissues (Figure 1).^19^

For 60 years, 5‐FU has been administered intravenously to treat cancer and still remains the backbone of many chemotherapy regimens for a range of solid cancers, including breast, gastrointestinal, pancreas, and head and neck. Its oral fluoropyrimidine cousin capecitabine is also commonly used for adjuvant treatment in the outpatient setting and is also metabolized by dihydropyridine dehydrogenase (DPD) inter alia. It is estimated that fluoropyrimidine therapy is administered to approximately 2 million patients globally annually.^20^ 5FU and capecitabine are included in the World Health Organisation Model Lists of Essential Medicines, so it is an important drug to know how to dose optimally. 5FU can be fatal if the exposure is excessive, and yet underdosing is associated with worse survival. To manage and individualize dosing to maximize efficacy and reduce toxicity, several international groups have provided guidance to clinicians regarding phenotype/genotype biomarkers and monitoring to guide dosing. For example, in France, the Cancer Institute and the health authority recommend measuring uracil concentration.^20^ They suggest alternative regimens without 5FU or capecitabine should be considered for uracil concentrations >150 ng/mL. The European Medicines Agency recommends DPD testing by assessing the presence only of the four main variants, the Dutch Working Group and the Clinical Pharmacogenetics Implementation Consortium (CPIC) have published similar recommendations, yet TDM is also recommended where there is concern around dose adjustments due to intermediate genetic results (i.e., DPD heterozygotes).^8^, ^9^, ^10^ CPIC specifically noted that *“to avoid underdosing of patients with genotype‐based dose reductions …follow‐up therapeutic drug monitoring is recommended”* (CPIC,^9^, ^10^ reviewed in Ref. [20]). In the Table of Pharmacogenetic Associations in the US Guidance, the FDA states for DPD deficiency that “insufficient data are available to recommend a dosage in intermediate metabolisers…”.^11^ This is similar to the EMA advice states “reduced starting dose should be considered in patients with identified partial DPD deficiency” but does not state how much reduction is needed; a crucial gap considering exposure is key for efficacy outcomes. Yet recommendations for halving or less of the dose for this group, despite their heterogeneity, are made by CPIC and followed by some centers. In Australia, 50% of dose recommendations for the heterozygotes were made in response to a chemotherapy death despite the fact it has been unknown what the DPD status of the patient who died was and further, it was unclear whether knowing the status would have prevented the death. The risks of underdosing or dropping the fluoropyrimidine as a response to a putative positive test may have also had effects on clinical outcomes as noted by the expert reviewer^21^, ^22^ and consistent with the evidence.^29^, ^36^

Many factors can modulate 5‐FU activity and its catabolism. The most described parameter is DPD deficiency although there are other genes involved in 5FU metabolism or activity that are likely relevant, for example, methyl tetrahydrofolate reductase (MTHFR). Four DPD variants are currently screened in clinical practice where guidelines have been implemented. These are DPYD*2A or rs3918290; DPYD*13 or rs55886062; c.2846A>T or D949V or rs67376798; HapB3 or rs75017182 or rs56038477.

However, measurement of these four only can miss cases of DPD deficiency, and mutations of other genes involved in 5FU metabolism and effect.^23^, ^25^, ^27^, Thus, there is a false reassurance issue if the screen comes back as negative for these mutations. Examining other laboratory data, it also appears likely that other functional variants exist that affect DPD activity and the pharmacokinetics and pharmacodynamics of fluoropyrimidines such as 5FU.^24^, ^25^, ^26^, ^27^ There is accumulating evidence that additional variations in genes are involved in fluoropyrimidine pharmacokinetics and pharmacodynamics which also increase the risk of toxicity.^28^, ^29^ A recent review proposed that a polygenic algorithm may be required.^35^

The issue of using only the four variants only for 5FU dose guidance highlights a mortality risk. If the dose is reduced because the screen is positive, the resulting 5FU exposure may be lower than the therapeutic range.^13^, ^16^ Although not examined in this study, others have shown that low 5FU exposure leads to worse survival.^30^, ^31^ In another study of colorectal cancer patients with 5FU toxicity, no DPD duplications or deletions were found, suggesting that the large genomic rearrangements in the DPD gene were not significant in the toxicity seen in 44.4% of the cases included in the study.^24^

Furthermore, the four variants chosen for routine screening are related to a policy of reducing 5‐FU‐induced toxicity in the 0.1% of homozygotes. However, the association between DPD *heterozygote* variants, risk of toxicity, and uracil levels or dihydrouracil: uracil (UH2/U) ratio are not clearly elucidated.^25^, ^27^, ^32^. Almost all literature on DPD is from European patients, although there is increasing literature on the prevalence of variants in non‐European descent patients or those of mixed descent, an increasingly large population. For example, in the white population, ~3%–7% have a DPD deficiency and 0.01%–0.1% have a complete deficiency^33^; around 8% of the African American population have a partial DPD deficiency.^34^ This is an additional risk to the introduction of genetics without any phenotype measurements as the relationship between gene expression and drug handling across these groups is currently unknown.^27^, ^35^, ^36^

#### 1.1.1 Tyrosine kinase inhibitors

Tyrosine kinase inhibitors (TKIs) are a large group of diversely structured compounds that have activity on a wide group of kinases. These have significant interindividual PK variability^37^ yet are often prescribed as a flat dose, even at the extremes of physiology and body size. It is well known that TKI exposure is influenced by many factors including patient physiology, pharmacogenetic disposition of metabolizing enzymes, distribution and excretion pumps, and kinase receptor gene sequences and expression, variability of which is large. Intraindividual genetic variability may also be affected by the intermittent expression of genes, affected by diet, treatments such as radiation or concomitant P450 inhibitory or inducing medicines, and timing of therapy. For example, dynamin‐independent and ‐dependent membrane kinetics are affected by a variety of medicines and can affect endocytosis and exocytosis, a key method of entry of TKIs and proteins into cells.^38^ Therefore, genetic testing for optimal drug dose needs to include kinetic and dynamic markers such as receptor biology that affect drug disposition and activity.^39^

In support of pharmacogenetics, reduced expression in the cytochrome P450 3A4 or co‐consumption of drugs that inhibit CYPP450 3A4 are known to both increase TKI exposure and improve cancer outcome, albeit with increased toxicity, and the reverse in patients who have high levels of CYP450 3A4 expression or co‐consumption of drugs that induce CYP450 3A4. These drugs are also substrates for drug transporters^27^ and thus it is difficult to know a patient's CYP status and other relevant genetic data, the degree of induction or inhibitionof enzyme systems are present, what the net genetic result is and what the degree of effect on protein expression is

Furthermore, genetic testing cannot measure patient characteristics such as adherence to treatment, a known impact on clinical outcome, nor environmental factors (food–drug and drug–drug interactions, including pharmacodynamic and other non‐P450 drug interactions).^40^ Yet, retrospective audits of TKIs in clinical practice have shown that achievement of targeted drug exposure correlates with treatment response with, for example, imatinib, nilotinib, dasatinib, erlotinib, sunitinib, and sorafenib.^41^, ^42^ The following table summarizes such points (Table 3).

**TABLE 3:** TKI pharmacogenetic testing.

Facts and issues that support gene testing to guide dose	Facts and issues that do not support gene testing to guide dose
Gene tests can be taken at any time.	Environmental factors, e.g., drugs, diet, as well as genetic factors can change P450 gene expression.
Does not need repeat monitoring after dose change.	Escudero‐Ortiz et al.^24^ in a retrospective analytical study involving patients on TKIs with TDM showed interindividual variability in the first cycle and in the last monitored cycle, 46.2% and 44.0% for erlotinib, 48.9% and 50.8% for imatinib, 60.7% and 56.0% for lapatinib, and 89.7% and 72.5% for sorafenib. Relationships between exposure and baseline characteristics were not statistically significant for any of the variables evaluated (weight, height, body surface area (BSA), age, and sex).
TKIs are mainly substrates for CYP3A4/5, so gene effects are predictable once the mutation is known.	Drugs that induce CYP3A4 activity (e.g., carbamazepine and dexamethasone) may decrease serum concentrations of CYP metabolized TKIs. And drugs that inhibit CYP3A4 activity (e.g., azole antifungals and calcium channel blockers) may increase serum concentrations of CYP‐metabolized TKIs. As well as P450, there are variants in ABCB1 or ABCB2 that could lead to altered TKI efflux and TKI response; the role of the drug importer OCT1, for example, is still controversial.
The evidence for TDM and its impact on clinical care of TKIs is consistent and broad across the class.	

It is interesting to review the literature and despite large amounts of quality pharmacological science supporting TDM with 5FU^43^ and TKIs, few sites globally routinely test exposure of these therapies in patients. Yet, there is significant interest in routine implementation of genotyping, a more novel test. However, even then, implementation of pharmacogenetic testing is varied—evidenced by how some hospital and international guidelines support genotyping and others do not. The question of how to ensure quality pharmacological science is debated and then implemented into practice. In this case, we ask readers to reflect on how much money has been directed by governments to genetic funding for pharmacology as opposed to phenotype funding. Also relevant to this discussion is the relative siloing of pharmacology teaching, research, and practice into different subdisciplines of cell biology, genetics, immunology, and similar. Departments have to compete for funding and build a novel science as opposed to working collaboratively to bring all aspects of the science together to support best practices for patients having chemotherapy. Ultimately, the best outcome for patient care is likely to involve genotyping and TDM working hand in hand with appropriate use of genotyping to identify potential issues with dosing and implementing TDM to optimize ongoing dosing.

## 2 CONCLUSIONS

The TAKE HOME MESSAGE for clinicians, pharmacists, and clinical pharmacologists in cancer dosing is as follows. Personalized medicine is:

- right drug,
- right decision (treat or not),
- right combination,
- right timing, and
- right dose.

…taking into account the clinical trial data for the patient group being treated AND finessing to *individual biological and pharmacological variables* either evident, or likely to be evident based on knowledge about comorbidity and body size. It is not a gene test without knowing the cancer biology, pharmacology, and pathophysiology behind it. It is a partnership where each genotype and phenotype test in collaboration with the patient and their clinical data can help make a personalized treatment decision.

Factors such as a patient's own dose–response curve and understanding where their biology, cancer biology, other cancer treatment, physiology, age, sex, comorbidity, diet, and other medications should inform the optimal use of genotype and phenotype tests in individualizing cancer dosing.

Further questions for clinicians and researchers working in the personalized medicine space in cancer include knowing which genes outrank others in terms of phenotype activity. Depending on the drug, are metabolic genes more important than membrane pumps, for example, and do all mutations in membrane pumps have a similar effect on drug disposition? How do we know how much drug is available (to bind to the receptor/enzyme)? What are the limitations of therapeutic drug measurements in the biological sample of the drug we are monitoring? Lastly, in this debate, the issue of what happens to gene predictions when there are multiple gene effects and multiple drugs in a chemotherapy combination is unclear. In the case of chemotherapy regimens using 5FU, assuming all toxicity in a multiple drug regimen can be informed by a gene test of four mutations in DPD is simplistic. Understanding the prognostic implications of underdosing on the first cycle or multiple dose adaptations is also unknown, as is the prognostic impact of the dose adaptation in response to upfront dose reduction based on an incomplete gene test. Our only clear knowledge is that TDM has clear outcome benefits for TKIs and 5FU, among others. Most but not all homozygotes for DPD deficiency have significant risk or mortality if the full dose of 5FU is given. Some of this variability in risk is likely related to diet (pyrimidine and/or folate intake) and other factors such as renal clearance and body composition. A future multiparametric approach may be the optimal way forward. In any case, it is clear that this area needs much further ongoing research.

## AUTHOR CONTRIBUTIONS

JM had the idea and pursued the hypothesis. JS helped with the literature review; AF added clinical literature and gave real world oncology examples and checked assumptions; PG gave laboratory insights into utility of phenotype and genotype testing.

## FUNDING INFORMATION

This research received funding as a Program Grant (PREDICT) from the Cancer Council NSW 2017.

## CONFLICT OF INTEREST STATEMENT

The authors declare no conflict of interest.

## ETHICS STATEMENT

No ethics approval was required.

## Data Availability

NA.
